# Chronic benzodiazepine prescriptions: for the better or for the worse?

**DOI:** 10.1192/j.eurpsy.2026.10386

**Published:** 2026-07-31

**Authors:** J. G. Bramness

**Affiliations:** Oslo University Hospital, Oslo, Norway

## Abstract

**Abstract:**

Chronic benzodiazepine (BZD) prescribing remains a debated issues in psychiatry, balancing clear short-term benefits against substantial long-term risks. This presentation reviews historical developments, current epidemiology, pharmacology, risks, and potential clinical roles for long-term BZD therapy, with a particular focus on emerging evidence and ongoing controversies.

Benzodiazepines replaced barbiturates in the mid-20th century because of their wider therapeutic window, rapid onset, dependable effects, and usefulness across anxiety, insomnia, and seizures. They remain widely prescribed across Europe, although use has declined over the last two decades, especially among younger patients. Prescription data show that BZD and Z-hypnotic use increase sharply with age, and that a substantial proportion of the population—particularly older adults—receive at least one annual prescription.

Despite therapeutic benefits, long-term BZD use carries well-documented risks. These include psychomotor impairment, cognitive decline, falls and fractures, paradoxical anxiety and insomnia, dependence, and interactions with alcohol or opioids that markedly increase overdose mortality. In schizophrenia, concomitant BZD treatment has been associated with elevated mortality rates (Tiihonen et al., 2011). Evidence regarding dementia risk is mixed, and tapering studies in psychogeriatric populations show limited cognitive reversibility after cessation.

Nevertheless, some patients may benefit from stable, long-term use, particularly those with treatment-resistant anxiety disorders, severe insomnia, epilepsy, or complex psychiatric comorbidities. Recent findings suggest that forced, rapid discontinuation can increase emergency visits and mortality (Maust et al., 2023), supporting a more individualized approach. This tension highlights the need for nuanced prescribing: minimizing unnecessary initiation, but recognizing that long-term therapy may be appropriate in selected cases.

Emerging clinical strategies include substitution programs—such as an ongoing Norwegian trial replacing illicit, high-potency BZDs with diazepam or oxazepam in opioid-agonist treatment—to reduce harm in high-risk populations.

The presentation concludes with practical guidance for prescribers: prioritize non-pharmacological treatments, avoid out-of-hours initiation, document clear treatment plans, schedule regular reviews, and closely monitor risk factors such as driving, polypharmacy, and signs of dependence. Prescribers’ own habits—including age-related differences in prescribing patterns—also shape national trends.

**Disclosure of Interest:**

None Declared

